# Use of Virtual Reality Techniques to Rehabilitate Military Veterans with Post-Traumatic Stress Disorder (Review)

**DOI:** 10.17691/stm2023.15.1.08

**Published:** 2023-01-28

**Authors:** M.G. Volovik, A.N. Belova, A.N. Kuznetsov, A.V. Polevaia, O.V. Vorobyova, M.E. Khalak

**Affiliations:** Leading Researcher, University Clinic; Privolzhsky Research Medical University, 10/1 Minin and Pozharsky Square, Nizhny Novgorod, 603005, Russia; Professor, Head of the Medical Rehabilitation Department; Privolzhsky Research Medical University, 10/1 Minin and Pozharsky Square, Nizhny Novgorod, 603005, Russia; Head of the Laboratory of Immersive and Remote Technologies; Privolzhsky Research Medical University, 10/1 Minin and Pozharsky Square, Nizhny Novgorod, 603005, Russia; Junior Researcher, University Clinic; Privolzhsky Research Medical University, 10/1 Minin and Pozharsky Square, Nizhny Novgorod, 603005, Russia; Junior Researcher, University Clinic; Privolzhsky Research Medical University, 10/1 Minin and Pozharsky Square, Nizhny Novgorod, 603005, Russia; Associate Professor, Department of General and Clinical Psychology Privolzhsky Research Medical University, 10/1 Minin and Pozharsky Square, Nizhny Novgorod, 603005, Russia

**Keywords:** post-traumatic stress disorder, PTSD, military veterans, virtual reality, VR, virtual reality exposure therapy, VRET

## Abstract

Post-traumatic stress disorder (PTSD) is a mental and behavioral disorder developing after a traumatic event like participation in combat activities. Objective diagnosis of combat PTSD and effective rehabilitation of war veterans is a current multifaceted problem with particularly high social costs. This review aims to evaluate the potential of virtual reality technique used as exposure therapy tool (VRET) to rehabilitate combat veterans and service members with PTSD.

The review was written following the guidelines of Preferred Reporting Items for Systematic Reviews and Meta-Analyses (PRISMA). The final analysis includes 75 articles published in 2017–2022.

VRET therapeutic effect mechanisms were examined along with protocols and scenarios of VRET combined with other interventions influencing PTSD treatment like pharmacotherapy, motion-assisted multi-modular memory desensitization and reconsolidation (3MDR), transcranial magnetic stimulation. The necessity is substantiated of psychophysiological measurements for objectification of PTSD clinical criteria and its dynamics during treatment. It was shown that inclusion of VRET to the package of PTSD rehabilitation interventions positively affects the results due to the enhanced effect of presence and greater experience personalization. Thus, VRET may be an effective, controlled, and cost-effective alternative for PTSD treatment in combatants, including those not responding to conventional therapy.

## Introduction

Post-traumatic stress disorder (PTSD) is a mental and behavioral disorder developing after a traumatic event like participation in combat activities, a terrorist act, traffic accident, sexual assault, domestic violence, and other threats to person’s life and health [1]. Symptoms of PTSD may include disturbing thoughts, feelings, or dreams related to the traumatic event; mental or physical distress due to cues associated with the trauma; avoidance of the event reminders; changes in thoughts or feelings and increased fight-or-flight response (Walter Cannon, 1932). A person with PTSD is at higher risk for suicide and intentional self-harm. These symptoms persist for more than a month after onset [2].

PTSD was first talked about after the Vietnam War; at the same time, development of a psychodiagnostic toolkit was started to identify the main symptoms making up the PTSD syndrome [2]. About 2.5 million U.S. servicemen were involved in operations in Iraq and Afghanistan, and according to various reports, 10−30% of them developed PTSD [3]. Studies show that modern wars veterans compared to veterans of previous eras are at increased risk of developing mental disorders, suicidal behavior, are prone to homelessness and problems with criminal justice [4]. According to a sample of U.S. veterans, $923 million were spent during two years to treat PTSD in the late 2010s putting a high burden on health care [5]. Long-term studies conducted for a year or more after trauma exposure allowed to reveal a typical symptom dynamic leading to either recovery or a prolonged PTSD treatment [6].

Considering that PTSD includes a wide enough spectrum of both somatic and psychogenic disorders, its diagnosis is rather difficult. The main goal is to rule out other conditions with different etiology. Then, the most important issue is to reveal the traumatic experience in the anamnesis and to establish the disorders connected with it [7]. The clinical questionnaires most commonly used to diagnose PTSD are: Structured Clinical Interview for DSM (SCID) — Davidson J., Smith R., Kudler H., 1989; Mississippi Scale for Post-Traumatic Stress Disorder — Keane T.M. et al., 1987, 1988; Clinical-administered PTSD Scale (CAPS) — Weathers F.W. et al., 1990; Impact of Event Scale-Revised (IES-R) — Horowitz M.J. et al., 1979; Combat Exposure Scale — Keane T.M. et al., 1989 [8].

Thus, PTSD diagnosis is primarily based on data obtained from the patient’s self-report. In this connection, factors allowing to establish a disorder presence and to assess its severity may depend on their conscious or unconscious misrepresentation in the patient’s verbal report. As a consequence, the necessity to objectivize PTSD clinical criteria in psychophysiological parameters space becomes more and more obvious. The results of a psychophysiological research confirmed diagnostically significant differences in functioning of the central and vegetative nervous system, sensory and cognitive systems between conditionally healthy people and people with PTSD [9]. Numerous instrumental data confirm differences in CNS functioning [10-12].

For instance, concerning PTSD course prognosis, contribution is discussed in detail of reduced parasympathetic tone and increased sympathetic activity measured applying various techniques of heart rate recording. Cognitive functions are assessed related to PTSD like memory, executive functions and cognitive flexibility. Emotional involvement and emotional regulation, risk and reward sensitivity are investigated using ECG, EEG, fMRI, and MRI along with eye tracking and photoplethysmography [13-17]. The development of these and other instrumental techniques is important for effective medical and psychological support of combat veterans at all stages of military service [14].

Conventional PTSD treatment is based on exposure therapy (ET), including prolonged exposure (PE), its success requiring presence of both the patient and physician at treatment sessions along with observing the protocol of activities. However, most of the necessary activities are performed without medical supervision outside the clinic.

It is well known that the rehabilitation process may become unnecessarily protracted if the patient loses interest in it or sufficient human and technical resources are unavailable [18]. Premature discontinuation of treatment was noted in 20–25% of veterans with PTSD [19]. Impaired affect regulation and finding treatment “too stressful” are among the cited reasons [17]. Thereby, service providers may need to adapt existing treatments to meet individual patient needs. An important problem is the risk of developing secondary traumatic stress among physicians and other personnel providing psychiatric care to military personnel, veterans, and persons who were in a conflict zone and faced traumatic situations daily [8, 20, 21].

Combining conventional rehabilitation techniques with the use of new technologies (e.g. brain-computer interfaces, noninvasive brain stimulators, wearable function analysis devices, etc.) may have a positive effect on recovery from PTSD [19]. Results of a 2018 meta-analysis by the International Society for Traumatic Stress Studies (ISTSS) showed that a number of novel therapies, e.g. neurobiological effects management and transcranial magnetic stimulation (TMS), evidenced effective therapy of persistent PTSD [22]. In 2020, ISTSS experts ranked virtual reality (VR) technology as a promising intervention for PTSD treatment [23].

Virtual reality is a form of human–computer interaction creating synthetic virtual environments to immerse users into them [24]. VR technologies for neural/ psychorehabilitation are relatively recent; technological advances and first scientific discoveries were soon followed by clinical implementation [18]. In this regard, evaluation of intervention effectiveness and changes in research priorities are reactive rather than proactive [25]. This stimulates a more intensive study of mechanisms of disorders and response control circuits, protocol refinements, improvement of research strategy, its material and technical resources.

**The aim of our review** is to analyze the possibilities of using virtual reality technologies in the rehabilitation of persons with post-traumatic stress disorder.

## Data search and analysis strategy

The review was written following the guidelines of Preferred Reporting Items for Systematic Reviews and Meta-Analyses (PRISMA) [26, 27]. A search was run of papers published during the last 5.5 years (2017–2021 and first half of 2022) in databases PubMed (Medline) (72 sources found) and Science Direct (112 sources found) using key words “post-traumatic stress disorder” or “PTSD” + “virtual reality” + “exposure therapy” + “military” or “veteran”. Sampling size was not restricted. Thematic publications were identified reviewing reference lists of relevant articles. The articles selection included three stages: 1) searching for articles in databases by key words and reading abstracts; 2) excluding papers by title or abstract and inclusion criteria; and 3) analyzing the full content of selected articles.

### Inclusion criteria

Articles in English were included into the study on analysis of VR hardware/software systems for psychotherapy of PTSD in service members and on discussion/evaluation of methods and results of diagnosis and/or therapy of PTSD in this group of patients. No Russian-language papers were found for 2017–2022 fully matching the given set of key words (“PTSD” + “VR” + “exposure therapy” + “combat veterans”).

### Exclusion criteria

Excluded were books; theoretical articles or secondary reviews; articles not in English; articles not emphasizing VR devices as a therapeutic intervention tool; studies not explicitly defining neurorehabilitation; studies published earlier than 2017.

After removing duplicates, the first three co-authors independently evaluated if the remaining papers in the list complied with their inclusion criteria. Thus, a total of 75 works were selected. Additionally, selected studies were used devoted to theoretical research within the investigated scope (see the Figure).

**Figure:**
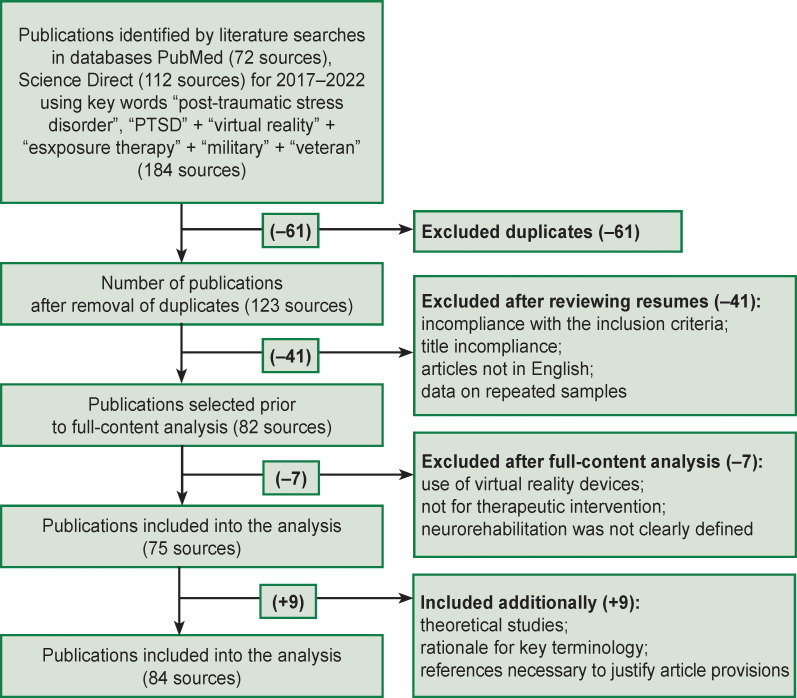
Structural diagram of literature search

## Mechanisms of VRET therapeutic effect

A major component of PTSD is the inability to suppress the maladaptive fear response resulting from pathologically increased activity in the amygdala and dorsal anterior cingulate cortex along with reduced activity in the ventromedial prefrontal cortex [1]. Increasing the endogenous activity of the latter, e.g. using TMS during fear-inducing stimuli processing, may be a promising method for inhibiting fear and improving treatment efficacy [28].

According to Liu et al. [29], virtual reality exposure therapy (VRET) is widely used to correct PTSD, phobic and anxiety disorders, nervous system disorders and to manage pain. VRET effectiveness relies on models of fear response formation based on origination of conditioned bonds anchoring the relevant pathological engrams; interruption of these bonds leads to fear reduction or suppression [30]. Re-activation of trauma memory with VRET allows to incorporate new information related to safety in therapeutic environment into patients’ cognitive patterns, thereby reducing fear and anxiety resulting from individual experience. This retraining is at the heart of the therapy and is an active process involving amygdala synaptic modification.

Landowska et al. [31] using a combination of functional near-infrared spectroscopy (fNIRS) and VR showed that both ET and VRET normalize brain activity in the circuit controlling fear. The authors measured the dynamics of oxygenated hemoglobin (HbO) concentration in the dorsolateral and ventromedial prefrontal cortex during several exposure sessions. Activity reduction was found in these areas during the first exposure; then, during three consecutive sessions, activity increased to normal contributing to inhibit amygdala activity and thus to retard the fear response.

Verger [32] conducted a study where therapy based on eye movement desensitization and reprocessing (EMDR) was combined with stress exposure to VR war scenes and reported a positive sign evidenced by improvement in precuneus metabolism (according to positron emission tomography) in servicemen with PTSD. After EMDR, connectivity reduction of metabolic changes between the precuneus and large bilateral clusters of cerebellum posterior areas (cerebellar lobules Crus I and VI) was observed concurrently with reduction of PTSD clinical manifestations severity. Increased metabolism in relevant cerebellum areas decreased after EMDR. Increased precuneus metabolic activity after EMDR therapy correlated with clinical improvement according to CAPS [33]. A study in mice described a neural pathway controlled by the superior colliculus that mediates persistent fear attenuation, when alternating bilateral sensory stimulation during EMDR is combined with conditioned stimuli [34]. Stimulation of that kind suppressed the activity of fear-encoding cells in the basolateral amygdala and stabilized inhibitory neurotransmission via a direct-coupled inhibition circuit from the mediodorsal thalamus. Apparently, this neural circuitry underlies an effective strategy for sustained attenuation of traumatic memories in PTSD.

Association was demonstrated between hypothalamic-pituitary-adrenal axis functioning and PTSD treatment outcome in veterans. In patients who responded to therapy, salivary cortisol levels before and after a VRET session (6 sessions) tended to be higher than average, this predicting greater improvement in PTSD symptoms [35, 36]. Higher cortisol levels associated with the session contribute to fear attenuation and reconsolidation and may be considered as biomarker of response to PTSD therapy.

## Protocols and scenarios of VRET combined with other PTSD therapies

VR exposure therapy is based on prolonged intervention aimed to virtually recreate the trauma patient story [37]. Protocols of PTSD therapy separate sessions and courses are available at a considerable number of studies [38–40, etc.]. Almost all researchers emphasize the importance of the meaningful part of the scenarios used for VRET.

The most widespread scenarios are VR narrative ones based on the patient’s life story presented by the patient from the narrator’s position [41]. The basic principles to develop war scenarios for VRET were defined with the participation of combat veterans with PTSD and should: be dynamic and include multisensory experience, be combined with conventional therapy, provide a sense of presence and realism of sensations [42].

VR exposure therapy can be effective to prevent PTSD before manifestation and to prevent PTSD augmentation after manifestation. Pyne et al. [43] used VRET in their study to run a preventive training course aimed at increasing soldiers’ resistance to occurrence of PTSD. Participants were divided into two groups: with heart rate variability biofeedback and with modification of cognitive distortions. Severity of PTSD symptoms was measured before manifestation, at 3 and 12 months after deployment. Overall, no significant effect of VRET was achieved in this study. Nevertheless, resistance training as a preventive measure against PTSD development was found to be useful because the subgroup of soldiers above 45 years had lower rates of PTSD symptoms after its formation compared to younger soldiers and to a control group having never been in a conflict zone.

### VRET combined with pharmacotherapy

Results of combined use of VRET and therapy with drugs like D-cycloserine and dexamethasone are presented in literature.

Rauch et al. [39] studied the change of SUDs scores (Subjective Units of Distress scale) during exposure to VRET and concurrent administration of D-cycloserine, alprazolam, or placebo. It was demonstrated that addition of D-cycloserine, considered to be a “cognitive enhancer” and to facilitate fear extinction, resulted in a less evident improvement compared to ET without addition of this drug [44]. During a study with participants randomly assigned to treatment groups (VRET/ET + D-cycloserine (50 mg)/placebo), Difede et al. [45] obtained results showing that D-cycloserine may enhance the PTSD response to both ET and VRET (3 months after a course of 9 sessions), and the improvement may be more significant in the latter case.

According to Maples-Keller et al. [46], some translational researches showed that dexamethasone administration can contribute to fear extinction in animal and human conditioning models. In their study, the authors found a reduction of PTSD symptoms in veterans after VRET, but no significant additional effect was found from taking dexamethasone. And dexamethasone improved no ET results. Besides, its intake was associated with frequent withdrawal from therapy (76.9% compared with 28.5% in the placebo group) [46]. The results of this study demonstrated the potential risks of transferring neurobiological models to the clinic.

The studies on odorants efficacy in VRET should also be noted. For instance, new ways of using aromatic agents for PTSD therapy and to prevent its development have been proposed to explore olfactory VR potential [47]. Late positive potential (LPP) amplitudes in response to VRET olfactory stimuli were studied to assess individual differences of emotional reactivity in combat veterans with PTSD. In the PTSD group, PTSD scores higher than normal were found to be associated with increased reactions to odors of diesel fuel and rotten eggs, but not n-butanol [15].

### VRET combined with motion-assisted, multi-modular memory desensitization and reconsolidation

It was suggested that one reason for poor response to PTSD treatment is persistent behavioral and cognitive avoidance of trauma reminders. Personalized VR combined with multi-sensory input and multi-modular memory desensitization and motion-assisted, multi-modular memory desensitization and reconsolidation (3MDR) was proposed to optimize therapy. 3MDR is an extended version of the EMDR method, which is considered one of the most effective in PTSD psychotherapy [36]. 3MDR facilitates memory retrieval, activates participation and increases attention during sessions, and multisensory input (like images and music) personalizes the experience [48] and increases emotional engagement. Evidence exists of physical activity positive effect on fear extinction as compared to sitting. This effect occurs as the patient moves in a virtual environment toward images of traumatic events of his/her own choice. It is assumed that 3MDR effectiveness depends on approaching behavioral response, as opposed to the avoidance response usually assumed by patients, and to strengthened divergent thinking [12].

3MDR protocols include projection of specially constructed tunnels for passage and patient self-selected digital images evoking memories of the traumatic event which are integrated into the software used for treatment [49]. A study of Jones et al. [38] showed that VR combined with 3MDR are effective for war veterans resistant to PTSD therapy. Positive effects were described on emotional dysregulation with cognitive-motor stimulation, narration, divergent thinking, re-evaluation of aversive stimuli, double processing and reconsolidation of traumatic memories [50].

Despite that 3MDR is considered a promising treatment, reduction of PTSD symptoms is not observed in everyone [49, 51]. To accurately assess the method effectiveness, analysis of biomarkers is required to confirm symptoms attenuation along with differently designed studies [52].

Being a complex and insufficiently studied intervention with unclear mechanism, the 3MDR method has significant limitations [21]. Adaptation of 3MDR to other forms of mobility, e.g. a wheelchair ergometer, is needed allowing to use the method for treating victims with walking disabilities.

### VRET combined with transcranial magnetic stimulation

Protocols were developed for simultaneous and sequential application of TMS and VRET, with the former considered preferable because during immersion in a contextually rich and immersive virtual environment associated with the fear emotion, subthreshold TMS allows to modulate the internal neural activation triggered by fear response. In the group with TMS and VR simultaneous application, reduction of galvanic skin response (GSR) between sessions significantly correlated with a clinically considerable reduction of PTSD symptoms severity [28, 40].

## Psychophysiological methods of VRET effectiveness assessment

Psychophysiological measures can provide important additional data for studying VRET responses variability. Psychophysiological methods of autonomic nervous system research are used to monitor the process of PTSD treatment with VRET, to predict and objectively evaluate the results [20, 45, 47, 53]. For instance, Gramlich et al. [6] recorded heart rate (HR), GSR, and skin temperature every 5 min during VR sessions, comparing the results with the clinicians’ assessment and self-questionnaire data. During the course of treatment, ET group participants showed greater increase of HR compared to the VRET group. GSR rates increased during early sessions; then, a habituation response was formed. Exposure to traumatic memories increased GSR, this correlating with PTSD symptoms reduction in the ET group.

Maples-Keller et al. [54] studied the dynamics of psychophysiological measures before and after VRET in patients with high and low levels of PTSD who responded to treatment. The authors recorded wincing, HR, and GSR during a VR demonstration of three standard trauma-related scenes. Trauma-induced wincing response to stimulation was observed in all patients before treatment. However, wincing in individuals with high response stopped after treatment. HR reactivity data evidenced treatment efficacy in both groups; no significant change of GSR reactivity was observed.

Measurement results were examined of GSR in response to standardized VR representation of non-personalized combat-related events (e.g., contact with the enemy, explosions) compared to non-combat events. Veterans with PTSD, in contrast to veterans without this disorder, showed higher GSR reactivity during combat operations in VR, which was not observed during peaceful events [55].

Park et al. [56] observed hyperactivity of sympathetic responses and increased hemodynamic reactivity in veterans with PTSD during both combat and noncombat-related mental stress during VRET along with impaired sympathetic and cardiovagal baroreflex sensitivity and increased inflammation (blood samples were analyzed for inflammatory biomarkers, C-reactive protein levels). However, the 1-minute cold pressor test applied showed no significant difference of sympathetic response, blood pressure, and HR between the groups with and without PTSD. VRET was particularly effective in reducing anxiety by eliciting reactions to realistic frightening stimuli in patients with PTSD, and this helped to eliminate cognitive and functional disorders [57].

Psychophysiological reactions were studied during 3MDR synchronized with VR environment [52]. Participants’ HR increased slightly during the sessions averaging below 100 bpm, while breathing became significantly more frequent averaging 40–50 breaths per minute. SUDS scores were very high at therapy beginning, this being consistent with the strong negative feelings experienced by the study participants during the sessions. However, during treatment, SUDS scores and negative feelings decreased, and positive feelings increased significantly coinciding with reduction of PTSD symptoms as assessed by clinicians. A strong psychophysiological response evidenced high emotional involvement during therapy.

In a number of studies, the participants’ state was examined using larger scale lab and instrumentation resources in addition to psychophysiological measurements. For instance, to assess the occurrence of psychological and psychophysiological problems in personnel (therapists and operators) who provided 3MDR + VRET therapy courses to veterans, multi-omics biomarkers (proteomic and genomic profiles of blood and saliva, neuroendocrine, immune-inflammatory mediators and microRNA) were studied, eye tracking, EEG, HR, breathing movements, and walking patterns were analyzed [21, 58]. 3MDR operators and physicians had no serious issues. Inclusion of genetic and psychobiological measures into predictors of response to treatment were the advantages of other study design [45].

In addition to autonomic measures, a number of articles cite positron emission tomography data on improvement of cerebellar and precuneus metabolism (following therapy) [32, 33], as well as of cardiovascular function [59].

## Efficiency, limitations and prospects of VRET

Currently, ET is the only intervention for PTSD with sufficient evidence of effectiveness [45]. In 2001, Rothbaum et al. [60] published the first open clinical trial showing a significant reduction of PTSD symptoms after VRET. In randomized clinical trials, conventional ET is most commonly used to actively control efficacy of VRET for PTSD in military personnel. No significant advantages of VRET over ET were found [61-63].

In studies of Beidel’s group [64, 65], individual VRET was compared to conventional group psychotherapy in an intensive outpatient format. PTSD symptoms, sleep quality, levels of depression, anger, guilt, and social isolation were assessed after treatment, after 3 and 6 months of follow-up. VRET contributed to a significant reduction of symptoms, and 65.9% of combatants no longer met diagnostic criteria for PTSD. However, VRET gave no optimal treatment outcomes for all problems associated with PTSD. Reduction of depression and anger was significant, but none of the interventions improved sleep, and reliable reduction of social isolation occurred only in those participants who received group therapy. Positive effects of treatment persisted for 6 months of follow-up.

As a secondary result of VRET compared to the control group (no treatment), increase of perceived social support was noted, this being one of the possible ways to reduce PTSD symptoms [66]. VRET effects concerned mainly depressive symptoms, with dose– response relationship observed: the greater the number of VRET sessions, the more significant improvement in patients’ state was recorded. A sustained reduction of PTSD symptoms was demonstrated at 3 and 6 months after VRET course completion [67]. A meta-analysis also showed that VRET is more effective than no treatment at all (waiting list control groups), while no significant difference was found between VRET and conventional therapy [61].

However, current advances in VR technologies demonstrate their potential to address cognitive and functional disorders in patients with PTSD. A key aspect of the therapeutic process in VRET is re-experiencing traumatic events affecting three clusters of symptoms: freezing, avoidance, strong arousal [68]. Increasing attention is being paid to VRET effects on selected PTSD aspects and the factors determining its effectiveness, along with using VR to predict PTSD symptoms development in veterans. This is especially true for persons resistant to conventional therapy.

VR exposure therapy as a digital method of delivering therapy to war veterans was more highly rated than the inconsistent results of cognitive-behavioral approaches. Being as effective as face-to-face delivery, VR reduces stigma and cost while increasing therapy access [69].

VRET was demonstrated to reduce the risk of cardiovascular disease associated with PTSD, including distant periods after treatment [59]. VRET was reported to be effective in distracting from fear to make movements related to possible pain (kinesiophobia) [53, 70].

Various symptoms of PTSD may change inequivalently over the course of treatment. Changes in re-experiencing symptoms in war veterans were found to precede changes in other PTSD criteria during VRET [71]. When comparing PE, VRET, and untreated groups of veterans with PTSD, an interdependent change in symptoms was shown. The authors suggested that exposures *in vivo* are more closely related to changes in overall PTSD symptoms than imaginal exposures in VRET [72].

Compared to a waiting list control group (untreated), VRET showed significantly better results for PTSD symptoms and symptoms of depression, but not symptoms of anxiety. Besides, no significant differences were found between the effectiveness of VRET and pharmacotherapy by the end of treatment [5].

A study of Loucks et al. [73] assessed PTSD, depression, and psychophysiological measures of distress before treatment, immediately after treatment, and after 3 months of follow-up. Significant improvement of the patients’ state was observed, and the percentage to initial number of participants meeting PTSD criteria continued to decrease: to 53% immediately after treatment, and to 33% after 3 months.

Heterogeneity of response to treatment by dynamics of 18 baseline variables was investigated in two groups of veterans: with best response to PE and with best response to VRET. It was found that significant factors of greater PTSD symptoms reduction in VRET were age (more effective in young people), no antidepressants taking, more evident symptoms of hyperarousal in PTSD and suicide risk above a minimum [74]. PE/VRET reduced suicidal ideation likelihood after treatment [75]. Thus, prescribing therapy based on individual patient profiles may significantly increase the effectiveness of combat-related PTSD treatment.

A study of Reger et al. [76] showed that anxiety symptoms associated with simulation in VRET are side effects of being in VR. The authors recommend to consider the possibility of increased anxiety levels and to interpret VRET results cautiously.

Stigma and attitudes toward mental health services were assessed before randomization and after 5 and 10 VRET sessions. After therapy, the soldiers showed a significant improvement in their openness to talking about mental health issues compared to those on the waiting list. Reduction of PTSD symptoms and positive changes in attitudes toward treatment seem to predict reduction of stigma later on [77].

The ability of trauma-related multisensory stimuli to enhance emotional engagement is a benefit of VRET that may be crucial to clinical outcome. When comparing SUDS scores during the first imaginal exposure to PE or VRET, it was noted that soldiers with greater initial severity of PTSD symptoms had higher mean or peak distress. No differences were found between the PE and VRET groups in terms of SUDS at the first or last session, or in habituation between sessions. However, reduction per every 10 SUDS units (both mean and peak) from the beginning of the first session to the end of treatment was associated with a greater decrease in CAPS scores for both groups. No intergroup differences were found neither in these dynamics nor in SUDS/habituation dependence on severity of PTSD symptoms [78].

In a study of Shulman et al. [79], part of active duty soldiers with PTSD in the PE and VRET groups were asked to participate in a preliminary survey about their intention to undergo a full course of treatment. Survey participants were less likely to drop out of the study compared to those who were not interviewed. The authors anticipate randomizing this phenomenon in future clinical trials.

Temporal relationships between clusters of PTSD symptoms in soldiers assessed by clinicians during treatment using PE, VRET, and a waiting list control state (untreated group) were studied [80]. No significant differences were found between PE and VRET groups, but compared to controls, these groups showed an earlier reduction of avoidance/stupor symptoms followed by a reduction of hyperarousal symptoms. The results of this study confirmed the effectiveness of any form of ET on PTSD symptoms, especially avoidance.

VR increasing availability associated with advances in mobile device technology allowing to track motions and to project images makes VRET a promising tool that can be used at work, at home, or on the go [81]. A veteran student who underwent cell-phone VRET reported decreased psychological symptoms of social anxiety and improved sleep quality after completing the course [82].

A group of researchers led by Rizzo [24] presented a VR application for treatment of servicemen and veterans with combat PTSD in the form of virtual environment BraveMind with an extension of simulations to 14 different therapeutic scenarios for PTSD assessment and prevention. The advantages of this therapy are interactivity, controllability, confidentiality, and possibility of personal adjustment. This allows for meaningful success in cases of resistant PTSD in veterans [83].

VR exposure therapy is used as a tool allowing to predict development of PTSD symptoms in veterans [44]. Patients with combat PTSD, in contrast to veterans without psychophysiological arousal, in response to combat stimuli, exhibited higher GSR reactivity during non-personalized and standardized combat presentation in VR, but not during peaceful events [55].

In their study, van Gelderen et al. [36] investigated the efficacy of combining VR with 3MDR to treat veterans with therapy-resistant PTSD (an average of 4 treatment failures in the past) compared to a control group receiving a nonspecific treatment component. Clinical symptoms of PTSD were assessed at the beginning and at the end of therapy, after 12 and 16 weeks of follow-up. After 3MDR, there was a more evident reduction of PTSD symptoms severity from initial level compared to the control group. High participant engagement was evidenced by low dropout rate (7%) with 45% of patients in the 3MDR group experiencing clinical improvement. However, no reliable differences between the groups in the long-term period are available, and this limits the conclusions about the effectiveness of the proposed technique and emphasizes the need for additional tests.

The subjective opinion was studied of veterans with therapy-resistant PTSD on processes and effects of VR + 3MDR treatment, along with dependence of PTSD symptoms improvement on their experience. When veteran-approved treatment processes were incorporated, distress was regulated, they felt supported, faced traumatic memories, allowed emotions to manifest, and disconnected themselves from the trauma. Veterans reported positive changes after 3MDR, including openness, new learning, self-understanding, closure and reintegration; they reported that 3MDR therapy allowed them to break through avoidance and to increase engagement, thereby making it easier to find and process traumatic memories [36].

It was shown that VRET with arousal control is effective to reduce severity of combat PTSD symptoms in untreated veterans in the first years after their return from war [3].

A regression meta-analysis of 30 studies with 1057 participants (including 5 PTSD studies) allowed to find that larger sample sizes were associated with smaller VRET effects compared to controls [84].

Uncertainty of meta-analytic evaluations [67] and insufficient data on whether digital treatment for PTSD in military personnel and war veterans leads to the same results as face-to-face treatment [69] limit the use of VRET. Issues of risk, safety, confidentiality, and potential harm (e.g., suicidal tendencies, avoidance potential) also require further consideration.

It should be noted that treatment leads to PTSD symptoms improvement not for all veterans [51, 62], therefore analysis of failure reasons is needed in each case. The results obtained in combat veterans may not be applicable to non-military veterans or to PTSD in civilians (more research is needed).

Weaknesses of VRET technologies include the use of non-validated self-report applications, difficulty and infrequency of staff communication with patients during PTSD deployment, low severity of PTSD symptoms throughout the study [36], and limitation of the follow-up period to 3–6 months. Note that some participants may exhibit strong emotional response during the study or experience side effects related to cybersickness [49, etc.] The presence is highly recommended of a second research team member observing the participant during sessions, other than the person operating the VR environment [28]. The cost of procedures should also be taken into account: for instance, the use of VR combined with 3MDR dramatically increases labor content and cost of sessions.

## Conclusion

The ambiguity of the results of VRET studies in patients with PTSD results from relatively small (but increasing over time) number of studies and the variety of protocols and scenarios. Besides, VRET can be an effective alternative to existing methods and demonstrated more successful treatment of patients with PTSD who did not respond to previous therapy.

The task of future studies is to find out the dynamics of changes in PTSD symptom cluster at all stages of treatment with VRET within individual sessions, at the end of the course, and in the long-term period.

## References

[1] Beberashvili Z., Javakhishvili J., Tabaghua S. (2021). Nature of trauma and pathways to healing authors..

[2] (2013). Diagnostic and Statistical Manual of Mental Disorders (DSM-5)..

[3] Wood D.P., Roy M.J., Wiederhold B.K., Wiederhold M.D. (2021). Combat-related post-traumatic stress disorder: a case report of virtual reality graded exposure therapy with physiological monitoring in a U.S. navy officer and a U.S. army officer.. Cureus.

[4] Pajak A. (2020). Special needs of and promising solutions for incarcerated veterans of Operation Enduring Freedom, Operation Iraqi Freedom, and Operation New Dawn.. J Correct Health Care.

[5] Kothgassner O.D., Goreis A., Kafka J.X., Van Eickels R.L., Plener P.L., Felnhofer A. (2019). Virtual reality exposure therapy for posttraumatic stress disorder (PTSD): a meta-analysis.. Eur J Psychotraumatol.

[6] Gramlich M.A., Smolenski D.J., Norr A.M., Rothbaum B.O., Rizzo A.A., Andrasik F., Fantelli E., Reger G.M. (2021). Psychophysiology during exposure to trauma memories: Comparative effects of virtual reality and imaginal exposure for posttraumatic stress disorder.. Depress Anxiety.

[7] Bukhtiyarov I.V., Glukhov D.V. (2018). Posttraumatic stress disorder formation in military officers in combat circumstances.. Meditsina truda i promyshlennaya ekologiya.

[8] Kharlamenkova N.E. (2017). Psychology of post-traumatic stress: results and prospects for future researches.. Psihologiceskij zurnal.

[9] Pineles S.L., Orr S.P., Nemeroff C.B., Marmar C. (2018). The psychophysiology of PTSD.. Post-traumatic stress disorder..

[10] Ge F., Yuan M., Li Y., Zhang W. (2020). Posttraumatic stress disorder and alterations in resting heart rate variability: a systematic review and meta-analysis.. Psychiatry Investig.

[11] Katz A.C., Norr A.M., Buck B., Fantelli E., Edwards-Stewart A., Koenen-Woods P., Zetocha K., Smolenski D.J., Holloway K., Rothbaum B.O., Difede J., Rizzo A., Skopp N., Mishkind M., Gahm G., Reger G.M., Andrasik F. (2020). Changes in physiological reactivity in response to the trauma memory during prolonged exposure and virtual reality exposure therapy for posttraumatic stress disorder.. Psychol Trauma.

[12] Nijdam M.J., Vermetten E. (2018). Moving forward in treatment of posttraumatic stress disorder: innovations to exposure-based therapy.. Eur J Psychotraumatol.

[13] Zelenina N.V., Nazarov S.S., Marchenko A.A., Rantseva S.A., Vypritskiy P.A., Yusupov V.V. (2019). Method for psychophysiological diagnosis of individual signs of chronic post-traumatic stress disorder in military combatants..

[14] Shamrey V.K., Marchenko A.A., Lobachev A.V., Tarumov D.A. (2021). Modern methods of mental disorders objectification in military service.. Social’naa i kliniceskaa psihiatria.

[15] Bedwell J.S., Bohil C.J., Neider M.B., Gramlich M.A., Neer S.M., OʼDonnell J.P., Beidel D.C. (2018). Neurophysiological response to olfactory stimuli in combat veterans with posttraumatic stress disorder.. J Nerv Ment Dis.

[16] Ben-Zion Z., Fine N.B., Keynan N.J., Admon R., Halpern P., Liberzon I., Hendler T., Shalev A.Y. (2019). Neurobehavioral moderators of post-traumatic stress disorder (PTSD) trajectories: study protocol of a prospective MRI study of recent trauma survivors.. Eur J Psychotraumatol.

[17] Hundt N.E., Ecker A.H., Thompson K., Helm A., Smith T.L., Stanley M.A., Cully J.A. (2020). “It didn’t fit for me:” a qualitative examination of dropout from prolonged exposure and cognitive processing therapy in veterans.. Psychol Serv.

[18] Freedman S. (2019). Virtual reality treatment for combat related PTSD: is Virtual Azza more effective than traditional exposure treatment?.

[19] Demkin A.D., Ivanov V.V., Kruglov V.I. (2019). New rehabilitation methods in the tratment of military personnel sterss disoreders in foreign armed forces.. Izvestia Rossijskoj voenno-medicinskoj akademii.

[20] Chaabane S., Etienne A.M., Schyns M., Wagener A. (2022). The impact of virtual reality exposure on stress level and sense of competence in ambulance workers.. J Trauma Stress.

[21] Jones C., Smith-MacDonald L., Van Veelen N., VanderLaan A., Kaneva Z., Dunleavy R.S., Hamilton T., Vermetten E., Bremault-Phillips S. (2022). Therapist and operator experiences utilizing multi-modal motion-assisted memory desensitization and reconsolidation (3MDR) for treatment of combat related posttraumatic stress disorder amongst military and veteran populations.. Eur J Psychotraumatol.

[22] International Society for Traumatic Stress Studies (ISTSS). (2018). ISTSS prevention and treatment guidelines..

[23] Forbes D., Bisson J.I., Monson C.M., Berliner L. (2020). Effective treatments for PTSD. Practice guidelines from the International Society for Traumatic Stress Studies (ISTSS)..

[24] Rizzo A., Roy M.J., Hartholt A., Costanzo M., Highland K.B., Jovanovic T., Norrholm S.D., Reist C., Rothbaum B., Difede J.A., Bowles S.V., Bartone P.T. (2017). Virtual reality applications for the assessment and treatment of PTSD.. Handbook of military psychology..

[25] Keshner E.A., Weiss P.T., Geifman D., Raban D. (2019). Tracking the evolution of virtual reality applications to rehabilitation as a field of study.. J Neuroeng Rehabil.

[26] Hutton B., Salanti G., Caldwell D.M., Chaimani A., Schmid C.H., Cameron C., Ioannidis J.P., Straus S., Thorlund K., Jansen J.P., Mulrow C., Catalá-López F., Gøtzsche P.C., Dickersin K., Boutron I., Altman D.G., Moher D. (2015). The PRISMA extension statement for reporting of systematic reviews incorporating network meta-analyses of health care interventions: checklist and explanations.. Ann Intern Med.

[27] Massetti T., da Silva, T.D., Crocetta T.B., Guarnieri R., de Freitas B.L., Bianchi Lopes P., Watson S., Tonks J., de Mello Monteiro C.B. (2018). The clinical utility of virtual reality in neurorehabilitation: a systematic review.. J Cent Nerv Syst Dis.

[28] van’t Wout-Frank M., Shea M.T., Larson V.C., Greenberg B.D., Philip N.S. (2019). Combined transcranial direct current stimulation with virtual reality exposure for posttraumatic stress disorder: feasibility and pilot results.. Brain Stimul.

[29] Liu Z., Ren L., Xiao C., Zhang K., Demian P. (2022). Virtual reality aided therapy towards Health 4.0: a two-decade bibliometric analysis.. Int J Environ Res Public Health.

[30] Maples-Keller J.L., Yasinski C., Manjin N., Rothbaum B.O. (2017). Virtual reality-enhanced extinction of phobias and post-traumatic stress.. Neurotherapeutics.

[31] Landowska A., Roberts D., Eachus P., Barrett A. (2018). Within- and between-session prefrontal cortex response to virtual reality exposure therapy for acrophobia.. Front Hum Neurosci.

[32] Verger A., Rousseau P.F., Malbos E., Chawki M.B., Nicolas F., Lançon C., Khalfa S., Guedj E. (2020). Involvement of the cerebellum in EMDR efficiency: a metabolic connectivity PET study in PTSD.. Eur J Psychotraumatol.

[33] Rousseau P.F., Malbos E., Verger A., Nicolas F., Lançon C., Khalfa S., Guedj E. (2019). Increase of precuneus metabolism correlates with reduction of PTSD symptoms after EMDR therapy in military veterans: an 18F-FDG PET study during virtual reality exposure to war.. Eur J Nucl Med Mol Imaging.

[34] Baek J., Lee S., Cho T., Kim S.W., Kim M., Yoon Y., Kim K.K., Byun J., Kim S.J., Jeong J., Shin H.S. (2019). Neural circuits underlying a psychotherapeutic regimen for fear disorders.. Nature.

[35] van Gelderen M.J., Nijdam M.J., de Vries F., Meijer O.C., Vermetten E. (2020). Exposure-related cortisol predicts outcome of psychotherapy in veterans with treatment-resistant posttraumatic stress disorder.. J Psychiatr Res.

[36] van Gelderen M.J., Nijdam M.J., Dubbink G.E., Sleijpen M., Vermetten E. (2020). Perceived treatment processes and effects of interactive motion-assisted exposure therapy for veterans with treatment-resistant posttraumatic stress disorder: a mixed methods study.. Eur J Psychotraumatol.

[37] Knaust T., Felnhofer A., Kothgassner O.D., Höllmer H., Gorzka R.J., Schulz H. (2020). Virtual trauma interventions for the treatment of post-traumatic stress disorders: a scoping review.. Front Psychol.

[38] Jones C., Miguel Cruz A., Smith-MacDonald L., Brown M.R.G., Vermetten E., Brémault-Phillips S. (2022). Technology acceptance and usability of a virtual reality intervention for military members and veterans with posttraumatic stress disorder: mixed methods Unified Theory of Acceptance and Use of Technology study.. JMIR Form Res.

[39] Rauch S.A.M., Koola C., Post L., Yasinski C., Norrholm S.D., Black K., Rothbaum B.O. (2018). In session extinction and outcome in virtual reality exposure therapy for PTSD.. Behav Res Ther.

[40] van’t Wout-Frank M., Philip N.S. (2021). Simultaneous application of transcranial direct current stimulation during virtual reality exposure.. J Vis Exp.

[41] Georgieva I., Georgiev G.V. (2019). Reconstructing personal stories in virtual reality as a mechanism to recover the self.. Int J Environ Res Public Health.

[42] Vianez A., Marques A., Simões de Almeida R. (2022). Virtual reality exposure therapy for armed forces veterans with post-traumatic stress disorder: a systematic review and focus group.. Int J Environ Res Public Health.

[43] Pyne J.M., Constans J.I., Nanney J.T., Wiederhold M.D., Gibson D.P., Kimbrell T., Kramer T.L., Pitcock J.A., Han X., Williams D.K., Chartrand D., Gevirtz R.N., Spira J., Wiederhold B.K., McCraty R., McCune T.R. (2019). Heart rate variability and cognitive bias feedback interventions to prevent post-deployment PTSD: results from a randomized controlled trial.. Mil Med.

[44] Bourla A., Mouchabac S., El Hage W., Ferreri F. (2018). e-PTSD: an overview on how new technologies can improve prediction and assessment of posttraumatic stress disorder (PTSD).. Eur J Psychotraumatol.

[45] Difede J., Rothbaum B.O., Rizzo A.A., Wyka K., Spielman L., Jovanovic T., Reist C., Roy M.J., Norrholm S.D., Glatt C., Lee F. (2019). Enhanced exposure therapy for combat-related Posttraumatic Stress Disorder (PTSD): study protocol for a randomized controlled trial.. Contemp Clin Trials.

[46] Maples-Keller J.L., Jovanovic T., Dunlop B.W., Rauch S., Yasinski C., Michopoulos V., Coghlan C., Norrholm S., Rizzo A.S., Ressler K., Rothbaum B.O. (2019). When translational neuroscience fails in the clinic: dexamethasone prior to virtual reality exposure therapy increases dropout rates.. J Anxiety Disord.

[47] Herz R.S. (2021). Olfactory virtual reality: a new frontier in the treatment and prevention of posttraumatic stress disorder.. Brain Sci.

[48] van Gelderen M.J., Nijdam M.J., Vermetten E. (2018). An innovative framework for delivering psychotherapy to patients with treatment-resistant posttraumatic stress disorder: rationale for interactive motion-assisted therapy.. Front Psychiatry.

[49] Bisson J.I., van Deursen R., Hannigan B., Kitchiner N., Barawi K., Jones K., Pickles T., Skipper J., Young C., Abbott L.R., van Gelderen M., Nijdam M.J., Vermetten E. (2020). Randomized controlled trial of multi-modular motion-assisted memory desensitization and reconsolidation (3MDR) for male military veterans with treatment-resistant post-traumatic stress disorder.. Acta Psychiatr Scand.

[50] Tang E., Jones C., Smith-MacDonald L., Brown M.R.G., Vermetten E.H.G.J.M., Brémault-Phillips S. (2021). Decreased emotional dysregulation following multi-modal motion-assisted memory desensitization and reconsolidation therapy (3MDR): identifying possible driving factors in remediation of treatment-resistant PTSD.. Int J Environ Res Public Health.

[51] van Gelderen M.J., Nijdam M.J., Haagen J.F.G., Vermetten E. (2020). Interactive motion-assisted exposure therapy for veterans with treatment-resistant posttraumatic stress disorder: a randomized controlled trial.. Psychother Psychosom.

[52] van Deursen R., Jones K., Kitchiner N., Hannigan B., Barawi K., Bisson J.I. (2021). The psychophysiological response during post-traumatic stress disorder treatment with modular motion-assisted memory desensitisation and reconsolidation (3MDR).. Eur J Psychotraumatol.

[53] Fowler C.A., Ballistrea L.M., Mazzone K.E., Martin A.M., Kaplan H., Kip K.E., Murphy J.L., Winkler S.L. (2019). A virtual reality intervention for fear of movement for Veterans with chronic pain: protocol for a feasibility study.. Pilot Feasibility Stud.

[54] Maples-Keller J.L., Rauch S.A.M., Jovanovic T., Yasinski C.W., Goodnight J.M., Sherrill A., Black K., Michopoulos V., Dunlop B.W., Rothbaum B.O., Norrholm S.D. (2019). Changes in trauma-potentiated startle, skin conductance, and heart rate within prolonged exposure therapy for PTSD in high and low treatment responders.. J Anxiety Disord.

[55] van’t Wout M., Spofford C.M., Unger W.S., Sevin E.B., Shea M.T. (2017). Skin conductance reactivity to standardized virtual reality combat scenes in veterans with PTSD.. Appl Psychophysiol Biofeedback.

[56] Park J., Marvar P.J., Liao P., Kankam M.L., Norrholm S.D., Downey R.M., McCullough S.A., Le N.A., Rothbaum B.O. (2017). Baroreflex dysfunction and augmented sympathetic nerve responses during mental stress in veterans with post-traumatic stress disorder.. J Physiol.

[57] Park M.J., Kim D.J., Lee U., Na E.J., Jeon H.J. (2019). A literature overview of virtual reality (VR) in treatment of psychiatric disorders: recent advances and limitations.. Front Psychiatry.

[58] Jones C., Smith-MacDonald L., Miguel-Cruz A., Pike A., van Gelderen M., Lentz L., Shiu M.Y., Tang E., Sawalha J., Greenshaw A., Rhind S.G., Fang X., Norbash A., Jetly R., Vermetten E., Brémault-Phillips S. (2020). Virtual reality-based treatment for military members and veterans with combat-related posttraumatic stress disorder: protocol for a multimodular motion-assisted memory desensitization and reconsolidation randomized controlled trial.. JMIR Res Protoc.

[59] Bourassa K.J., Stevens E.S., Katz A.C., Rothbaum B.O., Reger G.M., Norr A.M. (2020). The impact of exposure therapy on resting heart rate and heart rate reactivity among active-duty soldiers with posttraumatic stress disorder.. Psychosom Med.

[60] Rothbaum B.O., Hodges L.F., Ready D., Graap K., Alarcon R.D. (2001). Virtual reality exposure therapy for Vietnam veterans with posttraumatic stress disorder.. J Clin Psychiatry.

[61] Eshuis L.V., van Gelderen M.J., van Zuiden M., Nijdam M.J., Vermetten E., Olff M., Bakker A. (2021). Efficacy of immersive PTSD treatments: a systematic review of virtual and augmented reality exposure therapy and a meta-analysis of virtual reality exposure therapy.. J Psychiatr Res.

[62] McLay R.N., Baird A., Webb-Murphy J., Deal W., Tran L., Anson H., Klam W., Johnston S. (2017). A randomized, head-to-head study of virtual reality exposure therapy for posttraumatic stress disorder.. Cyberpsychol Behav Soc Netw.

[63] Meyerbröker K., Morina N. (2021). The use of virtual reality in assessment and treatment of anxiety and related disorders.. Clin Psychol Psychother.

[64] Beidel D.C., Frueh B.C., Neer S.M., Lejuez C.W. (2017). The efficacy of trauma management therapy: a controlled pilot investigation of a three-week intensive outpatient program for combat-related PTSD.. J Anxiety Disord.

[65] Beidel D.C., Frueh B.C., Neer S.M., Bowers C.A., Trachik B., Uhde T.W., Grubaugh A. (2019). Trauma management therapy with virtual-reality augmented exposure therapy for combat-related PTSD: a randomized controlled trial.. J Anxiety Disord.

[66] Bourassa K.J., Smolenski D.J., Edwards-Stewart A., Campbell S.B., Reger G.M., Norr A.M. (2020). The impact of prolonged exposure therapy on social support and PTSD symptoms.. J Affect Disord.

[67] Deng W., Hu D., Xu S., Liu X., Zhao J., Chen Q., Liu J., Zhang Z., Jiang W., Ma L., Hong X., Cheng S., Liu B., Li X. (2019). The efficacy of virtual reality exposure therapy for PTSD symptoms: a systematic review and meta-analysis.. J Affect Disord.

[68] Maples-Keller J.L., Price M., Rauch S., Gerardi M., Rothbaum B.O. (2017). Investigating relationships between PTSD symptom clusters within virtual reality exposure therapy for OEF/OIF veterans.. Behav Ther.

[69] Jones C., Miguel-Cruz A., Smith-MacDonald L., Cruikshank E., Baghoori D., Kaur Chohan A., Laidlaw A., White A., Cao B., Agyapong V., Burback L., Winkler O., Sevigny P.R., Dennett L., Ferguson-Pell M., Greenshaw A., Brémault-Phillips S. (2020). Virtual trauma-focused therapy for military members, veterans, and public safety personnel with posttraumatic stress injury: systematic scoping review.. JMIR Mhealth Uhealth.

[70] Fowler C.A., Ballistrea L.M., Mazzone K.E., Martin A.M., Kaplan H., Kip K.E., Ralston K., Murphy J.L., Winkler S.L. (2019). Virtual reality as a therapy adjunct for fear of movement in veterans with chronic pain: single-arm feasibility study.. JMIR Form Res.

[71] Holder N., Holliday R., Wiblin J., Surís A. (2019). Patterns and temporal precedence of symptom change during cognitive processing therapy for military sexual trauma-related posttraumatic stress disorder.. Behav Cogn Psychother.

[72] Norr A.M., Bourassa K.J., Stevens E.S., Hawrilenko M.J., Michael S.T., Reger G.M. (2019). Relationship between change in in-vivo exposure distress and PTSD symptoms during exposure therapy for active duty soldiers.. J Psychiatr Res.

[73] Loucks L., Yasinski C., Norrholm S.D., Maples-Keller J., Post L., Zwiebach L., Fiorillo D., Goodlin M., Jovanovic T., Rizzo A.A., Rothbaum B.O. (2019). You can do that?!: feasibility of virtual reality exposure therapy in the treatment of PTSD due to military sexual trauma.. J Anxiety Disord.

[74] Norr A.M., Smolenski D.J., Katz A.C., Rizzo A.A., Rothbaum B.O., Difede J., Koenen-Woods P., Reger M.A., Reger G.M. (2018). Virtual reality exposure versus prolonged exposure for PTSD: which treatment for whom?. Depress Anxiety.

[75] Norr A.M., Smolenski D.J., Reger G.M. (2018). Effects of prolonged exposure and virtual reality exposure on suicidal ideation in active duty soldiers: an examination of potential mechanisms.. J Psychiatr Res.

[76] Reger G.M., Smolenski D., Edwards-Stewart A., Skopp N.A., Rizzo A.S., Norr A. (2019). Does virtual reality increase simulator sickness during exposure therapy for post-traumatic stress disorder?. Telemed J E Health.

[77] Reger G.M., Bourassa K., Norr A.M., Buck B. (2020). The impact of exposure therapy on stigma and mental health treatment attitudes among active duty U.S. soldiers with combat related PTSD.. J Psychiatr Res.

[78] Reger G.M., Smolenski D., Norr A., Katz A., Buck B., Rothbaum B.O. (2019). Does virtual reality increase emotional engagement during exposure for PTSD? Subjective distress during prolonged and virtual reality exposure therapy.. J Anxiety Disord.

[79] Shulman G.P., Buck B.E., Gahm G.A., Reger G.M., Norr A.M. (2019). Effectiveness of the intent to complete and intent to attend intervention to predict and prevent posttraumatic stress disorder treatment drop out among soldiers.. J Trauma Stress.

[80] Stevens E.S., Bourassa K.J., Norr A.M., Reger G.M. (2021). Posttraumatic stress disorder symptom cluster structure in prolonged exposure therapy and virtual reality exposure.. J Trauma Stress.

[81] Jerdans S.W., Grindle M., van Woerden H.C., Boulos M.N.K. (2018). Head-mounted virtual reality and mental health: critical review of current research.. JMIR Serious Games.

[82] Trahan M.H., Morley R.H., Nason E.E., Rodrigues N., Huerta L., Metsis V. (2021). Virtual reality exposure simulation for student veteran social anxiety and PTSD: a case study.. Clin Soc Work J.

[83] Rizzo A.S., Shilling R. (2018). Clinical virtual reality tools to advance the prevention, assessment, and treatment of PTSD.. Eur J Psychotraumatol.

[84] Carl E., Stein A.T., Levihn-Coon A., Pogue J.R., Rothbaum B., Emmelkamp P., Asmundson G.J.G., Carlbring P., Powers M.B. (2019). Virtual reality exposure therapy for anxiety and related disorders: a meta-analysis of randomized controlled trials.. J Anxiety Disord.

